# An enhanced dynamic transmission opportunity scheme to support varying traffic load over wireless campus networks

**DOI:** 10.1371/journal.pone.0238073

**Published:** 2020-08-26

**Authors:** Zazilah May, M. K. Alam, Khaleel Husain, Mohammad Kamrul Hasan

**Affiliations:** 1 Department of Electrical and Electronic Engineering, Universiti Teknologi PETRONAS, Seri Iskandar, Perak, Malaysia; 2 Center for Cyber Security, Faculty of Information Science and Technology, Universiti Kebangsaan Malaysia, Bangi, Malaysia; Wuhan University, CHINA

## Abstract

Transmission opportunity (TXOP) is a key factor to enable efficient channel bandwidth utilization over wireless campus networks (WCN) for interactive multimedia (IMM) applications. It facilitates in resource allocation for the similar categories of multiple packets transmission until the allocated time is expired. The static TXOP limits are defined for various categories of IMM traffics in the IEEE802.11e standard. Due to the variation of traffic load in WCN, the static TXOP limits are not sufficient enough to guarantee the quality of service (QoS) for IMM traffic flows. In order to address this issue, several existing works allocate the TXOP limits dynamically to ensure QoS for IMM traffics based on the current associated queue size and pre-setting threshold values. However, existing works do not take into account all the medium access control (MAC) overheads while estimating the current queue size which in turn is required for dynamic TXOP limits allocation. Hence, not considering MAC overhead appropriately results in inaccurate queue size estimation, thereby leading to inappropriate allocation of dynamic TXOP limits. In this article, an enhanced dynamic TXOP (EDTXOP) scheme is proposed that takes into account all the MAC overheads while estimating current queue size, thereby allocating appropriate dynamic TXOP limits within the pre-setting threshold values. In addition, the article presents an analytical estimation of the EDTXOP scheme to compute the dynamic TXOP limits for the current high priority traffic queues. Simulation results were carried out by varying traffic load in terms of packet size and packet arrival rate. The results show that the proposed EDTXOP scheme achieves the overall performance gains in the range of 4.41%—8.16%, 8.72%—11.15%, 14.43%—32% and 26.21%—50.85% for throughput, PDR, average ETE delay and average jitter, respectively when compared to the existing work. Hence, offering a better TXOP limit allocation solution than the rest.

## Introduction

Recent enhancements in wireless networking technologies coupled with improvements in computing power and storage capabilities have widened the scope of interactive multimedia (IMM) applications. These applications are diverse from online video streaming [1–3] to multimedia messaging [4–6] and have different Quality of Service (QoS) requirements. Reliable transportation, high-quality data storage, and easy access are some of the major requirements of these emerging applications [7]. Hence, in order to enable these applications, a network that is adaptable to the different QoS requirements of the IMM traffic is required. Wireless campus networks (WCN) [8] typically defined as the interconnection of multiple wireless local area networks (WLANs) [9] are widely used by academia, industry and other various organizations to offer IMM applications. A popular medium access control (MAC) protocol usually deployed in WCN is distributed coordination function (DCF) [10] protocol of the IEEE 802.11 standard. With the ever-increasing demand for different wireless services, the provision of QoS differentiation has become a critical concern for future wireless IMM communication. Existing DCF protocol does not guarantee the QoS requirements of IMM traffic due to limited support for real-time services. To address this issue, an amendment to IEEE 802.11 standard known as IEEE 802.11e [11] was introduced that consists of a contention-based channel access protocol called enhanced distributed channel access (EDCA). EDCA offers service differentiation function by categorizing the traffic stream into four different access categories (ACs) namely voice, video, best effort, and background [11].

### Motivation

In EDCA, various parameters at the MAC layer are regulated in order to distinguish different ACs. One such parameter is transmission opportunity (TXOP) that decides the duration for which data packets are allowed to be transmitted through the channel without interruption. By default, the parameter values are fixed based on priorities of different ACs which works well when the traffic load remains the same [11]. However, when the traffic load varies, especially during high traffic load, the default EDCA protocol fails to provide guaranteed QoS for high priority IMM traffic due to fixed TXOP limits. Also, part of the bandwidth remains unused during low traffic load as the channel is occupied until the assigned TXOP limit expires even though there is no traffic flow to transfer. To overcome these issues, the dynamic TXOP allocation schemes have been introduced in [12–17]. To address this issue, an appropriate allocation scheme that dynamically adapts its TXOP limits based on varying traffic load needs to be developed.

### Existing work

A dynamic TXOP allocation scheme assigns the variable length of TXOP for the different types of traffic depending upon the current status of the traffic flows, wireless channel conditions, and pre-setting threshold values. Existing dynamic TXOP allocation schemes have been proposed in [12–17]. Work in [12, 13] proposes a threshold-based dynamic TXOP allocation scheme that based on the current status of traffic flow not only sets the minimum and maximum TXOP threshold values but also adjusts the TXOP limit dynamically within these ranges. However, in case of significant variation of the traffic load, especially when the pre-setting threshold values are lower or higher than the current traffic load, the existing system may result in degraded network performance.

### Key contributions

In this article, the focus is on the effective use of the TXOP parameter as a way of intra-AC QoS differentiation for IMM applications. Specifically, the article aims to enhance the existing threshold-based TXOP limit scheme by deploying the proposed extended dynamic TXOP (EDTXOP) mechanism. The key contributions of the work are as follows:

- Deployment of an enhanced threshold-based EDTXOP mechanism in EDCA protocol involving dynamic adjustment of TXOP limit based on the current queue size to improve the network throughput and packet delivery ratio (PDR) while also minimizing the average end-to-end (ETE) delay and average jitter for IMM applications.
- Analytical estimation of EDTXOP scheme taking into account the pre-setting threshold-based dynamic TXOP limit calculation at MAC layer for different high priority traffic queues.

## Related work

There exist considerable work on DCF [18, 19] and EDCA [12–16, 20–29] protocols. The majority of the work on EDCA protocol focused on the QoS differentiation parameters namely arbitrary inter-frame space (AIFS), contention window (CW), and TXOP. Here, AIFS is the minimum time interval a station has to wait after sensing the channel as idle before it can start its back-off timer. Furthermore, the CW parameter can be regulated between the range *CW*_*min*_ to *CW*_*max*_ to enhance the network uplink and downlink fairness. The small value of CW in case of low traffic conditions minimizes the waiting time and improves the global throughput. However, during the high load traffic condition, low values of CW increase the collision probability [30]. TXOP allows transmitting a burst of frames based on service differentiation through the wireless medium without re-entering any traffic from other stations. In addition, the TXOP parameter not only supports the service differentiation but, also improves the bandwidth utilization of the network. There exist considerable work [12–16, 25–29] on this parameter for IMM applications.

A threshold-based dynamic TXOP (TBD-TXOP) scheme is proposed in [13] to facilitate the intra-ACs QoS in EDCA. Here, the TXOP limit is adjusted dynamically depending upon the current queue length of IMM traffics and the pre-setting threshold values. In addition, an analytical model is developed to evaluate the performance of this scheme. TBD-TXOP although shows slightly better efficiency in intra-AC QoS differentiation during normal traffic conditions but, the scheme does not adapt well for varying traffic conditions. Low traffic load leads to wastage of channel bandwidth, while TBD-TXOP might be unable to provide QoS guarantee for high priority traffic in case of heavy traffic load. Another dynamic TXOP allocation (DTA) mechanism is presented in [16] that offers QoS for high priority IMM traffic in hybrid coordinated function channel access (HCCA) protocol. The TXOP limit the variable duration for different IMM traffic is assigned here based on the number of MAC Service Data Units (MSDUs) that are to be transmitted on the wireless channel. Simulations of the DTA scheme highlight the network performance improvements in terms of ETE delay, throughput, and PDR. Here, the maximum TXOP limit for an admitted traffic stream is expressed as follows:

$$\begin{array}{c} TXOP_{i}=[\{\frac{\left(N_{i}*L_{i}\right)}{R_{i}}+O\},(\frac{M}{R_{i}}+O)] \end{array}$$ (1)

where *i* is the index of quality station (QSTA), *N*_*i*_ is the number of arrived MSDUs in the QSTA, *L*_*i*_ is the nominal MSDU size, *M* is the maximum MSDU size, and *R*_*i*_ is the physical transmission rate. Here, *O* is the overhead and is calculated as follows:

$$\begin{array}{c} O=(SIFS*R_{i}) \end{array}$$ (2)

where SIFS is the short inter-frame space. The number of nominal MSDUs, *N*_*nMSDU*_, considers only one unit of overhead which is stated in Eq (3).

$$\begin{array}{c} N_{nMSDU}=\{\frac{\left(N_{i}*L_{i}\right)}{R_{i}}+O\} \end{array}$$ (3)

In Eq (2), the overhead is converted from a time-based unit to a size-based unit. This is the reason why Eq (3) can be written as in Eq (4) as follow:

$$\begin{array}{c} Q_{i}=\{(N_{i}*L_{i})+O\} \end{array}$$ (4)

Accordingly, the ACK and MSDUs header need to be considered to calculate the overhead *O*. Work in [31] introduced a distributed TXOP adaptation mechanism (DTAM) for the IEEE802.11e EDCA protocol where the TXOP limit is assigned based on the computed throughput. Here, each node calculates its throughput and compares it with a target value. If the calculated throughput is lower than the target value, the TXOP limit of the node is increased. In contrast, when the accounted throughput is higher than the target value, the node decreases its TXOP limit. The numerical results highlight the DTAM scheme’s global consistency while acquiring the target throughput of the network. However, it is mentioned in [31] that in case of very high target throughput, the network’s QoS requirements can neither be improved nor guaranteed even when the TXOP limit is set at the maximum value. In this case, the TXOP limit range needs to be extended and regulated between 32 *μ*sec and 8160 *μ*sec that is defined in the IEEE802.11e standard. A comprehensive analytical model of the contention-free bursting (CFB) scheme is presented in [32] with an aim to evaluate the ETE delay, throughput, and packets loss probability under unsaturated traffic condition. In addition, the impact of the number of stations and the TXOP limit parameter on the wireless network performance is also analyzed. Simulations validate the CFB model by showing its efficiency in enhancing the WLAN’s performance. It also highlights it as a cost-efficient tool to achieve QoS requirements for a wireless network by varying MAC parameter settings. However, one of the limitations of the CFB scheme is its resource allocation parameter which is fixed for specific traffic and is not suitable for varying network traffic conditions. Work in [17] proposed a contention-based EDCA protocol named dynamic TXOP mechanism (DTM) with an aim to upgrade the QoS and efficiency of the network in assigning the TXOP limit scheme dynamically. The TXOP limit allocation, *TXOP*_*lim*_, is computed as follows:

$$\begin{array}{c} TXOP_{lim}=N_{pq}*L_{p} \end{array}$$ (5)

where, *L*_*p*_ is known as the average length of packets computed as the duration needed for transmitting MSDU through PHY layer and *N*_*pq*_ is the number of packets in the queue, which is calculated every 100ms beacon interval of time. The simulation results demonstrated that the DTM improved the network performance and controlled the temporal saturation for high priority real-time traffics in the low loaded network. However, DTM does not consider the MAC overhead to assign the variable TXOP limit, which is significant in utilizing the channel bandwidth appropriately over the network. Distinguished from the current solutions for dynamic adjustment of the TXOP limit parameter issue, a threshold-based EDTXOP scheme is proposed. EDTXOP dynamically regulates the TXOP limit based on the current queue size of the same AC’s IMM traffic while also utilizing an appropriate MAC overhead.

## Materials and methods

In this section, the details regarding the mechanism, simulation setup and performance metrics utilized for the proposed work are explained.

### EDTXOP mechanism

**Fig 1:**
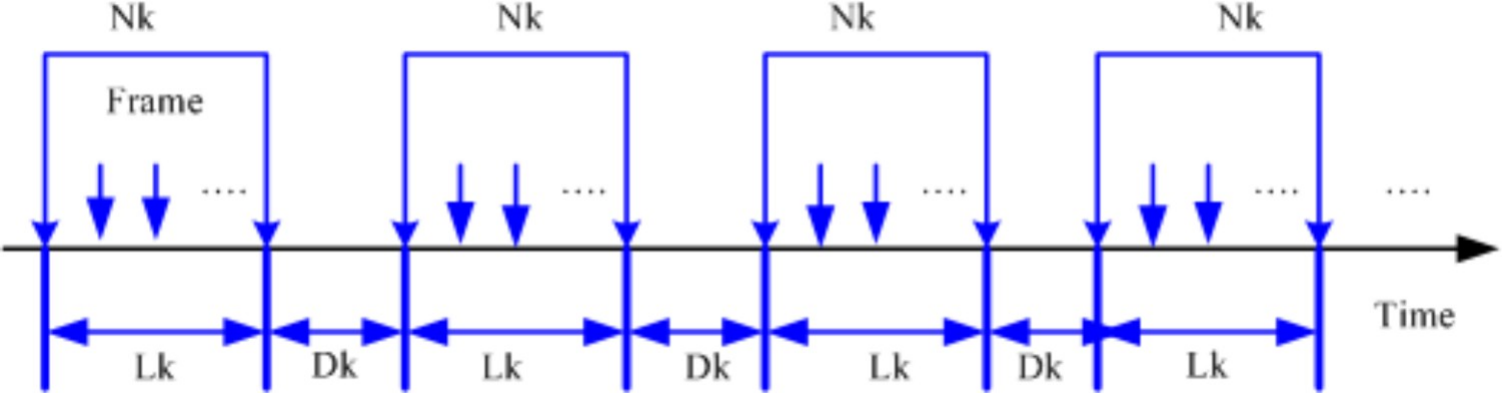
Traffic stream arriving pattern.

EDTXOP scheme addresses the above-mentioned limitation of the existing dynamic TXOP limit allocation mechanism [13, 16]. Specifically, the EDTXOP mechanism allocates the TXOP limit dynamically based on the number of frame sizes including an appropriate MAC overhead and the maximum transmitted physical data rate through the current channel for high priority IMM traffic over WCNs. The IMM traffic is considered as the constant bit rate (CBR) in the proposed scheme. The arriving traffic stream has been assumed to be in the MAC layer scheduler buffer waiting in the queue to get the permission to access the wireless channel. Fig 1 shows the proposed arriving pattern of the IMM traffic stream.

**Fig 2:**
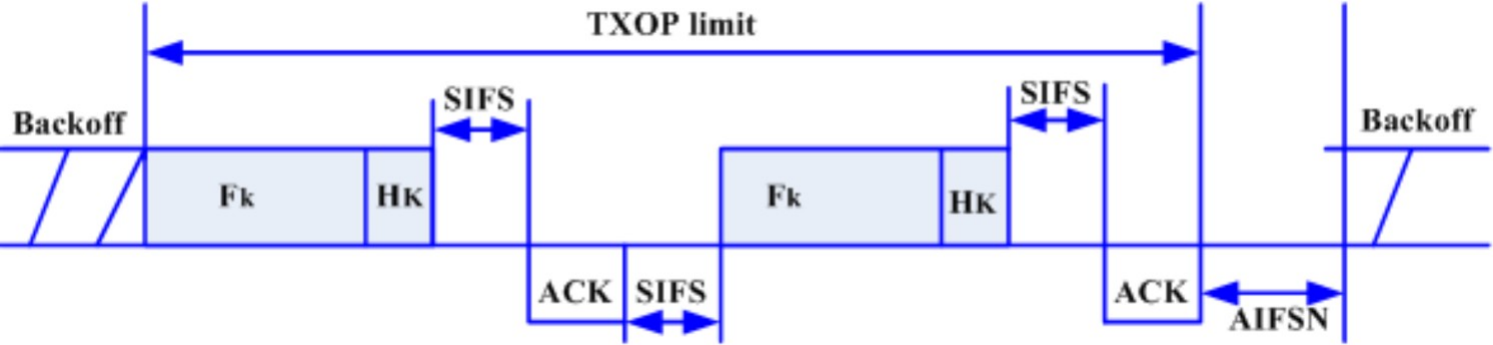
A sample of EDCA frame exchange sequence. Here, *F*_*k*_ is the MSDUs size and *H*_*k*_ is the MSDUs frame header.

As can be seen in the figure, *k* is assumed as the index of QSTA, *N*_*k*_ is the number of MAC Service Data Units (MSDUs), *L*_*k*_ is the length of the MSDUs, and the inter-arrival time is denoted as *D*_*k*_. The scheduler determines the TXOP limit period needed for each high priority traffic stream by considering the number of MSDUs and the length of the MSDUs which is included in the overhead of each MSDU. The quality access point (QAP) then assigns the TXOP limit duration sending the beacon frame through the QSTA for each traffic stream. Fig 2 shows a sample of the EDCA frame exchange sequence. As can be seen in the figure, the actual queue size of the traffic is considered as consisting of two parts, that is the data and the associated overhead. These two parts are used along with the number of frames to determine the queue size, which is then utilized in the TXOP limit estimation.

#### Analytical estimation of EDTXOP mechanism

In this section, an analytical estimation of the EDTXOP scheme is explained. The proposed EDTXOP mechanism estimates the TXOP limit according to two parameters. The first parameter is the current IMM high priority traffics queue with the same category, available in the MAC layer scheduler’s buffer while the second parameter is the pre-setting thresholds. The mathematical notations required for the analytical estimation of the EDTXOP scheme are highlighted in Table 1.

**Table 1:** The mathematical notations of the EDTXOP scheme.

*D*_*n*_	Expected packet delay	*t*_*s*_	Time stamp when the packet is generated at sender
*D*_*k*_	Inter arrival time	*N*_*r*_	Total number of packets received at the receiver
*F*_*k*_	MSDUs size	*N*_*s*_	Total number of packets sent from the sender
*H*_*k*_	MSDUs frame header	*S*_*bits*_	Size of each packet in bits
*i*, *k*	Index of the QSTA	*R*_*i*_	Physical transmission rate
*L*_*i*_	Nominal MSDU size	*R*_*k*_	Maximum physical data rate
*L*_*k*_	Length of the MSDUs	*R*_*n*_	Packet arrival rate
*M*	Maximum MSDU size	*Q*_*k*_	Total queue size
*N*_*pq*_	Number of packets in the queue	*TXOP*_*k*_	Current assigned TXOP limit
*N*_*i*_	Number of arrived MSDUs	Γ_*min*_	Minimum threshold
*O*	Overhead	Γ_*max*_	Maximum threshold
*t*_*c*_	Time taken for current packet to reach destination	*TXOP*_*max*_	Maximum TXOP limit
*t*_*p*_	Time taken for previous packet to reach destination	*TXOP*_*min*_	Minimum TXOP limit
*t*_*d*_	Time stamp when the packet reaches destination	*L*_*p*_	The average length of packets

The mathematical estimation of the TXOP duration is divided into two parts. The first part is the current size of MSDUs that are waiting to admit the channel while the second part is associated with the MAC overhead that appeared when accessing the wireless channel. Based on Fig 2, the overhead of the sequence is defined as follows:

$$\begin{array}{c} O=\{(SIFS*R_{k}+H_{k})+(ACK+SIFS*R_{k})\}, \end{array}$$ (6)

where *O* is the total frame overhead and *R*_*k*_ is the maximum physical data rate of the channel. Once the overhead is computed, the size of MSDUs can be expressed as follows:

$$\begin{array}{c} F_{k}=(L_{k}+O). \end{array}$$ (7)

Thereby, the total aggregated size of the queue, *Q*_*k*_, can be calculated by multiplying the number of packets in the queue with the size of the MSDUs as follows:

$$\begin{array}{c} Q_{k}=\{N_{pq}*F_{k}\}=(N_{pq}*L_{k})+(N_{pq}*O). \end{array}$$ (8)

From Eq (8), the TXOP limit for the traffic stream is expressed as follows:

$$\begin{array}{c} \begin{array}{c} TXOP_{k}=max\{(\frac{(N_{pq}*L_{k}}{R_{k}})+(\frac{N_{pq}*O}{R_{k}})\},\{(\frac{F}{R_{k}})+(\frac{O}{R_{k}})\} \end{array} \end{array}$$ (9)

where *F* is defined as the maximum MSDU.

#### EDTXOP limit allocation

The proposed EDTXOP mechanism can measure at each QSTA based on *Q*_*k*_ according to Eq (8). QSTA, upon the arrival of the frame, starts to estimate *Q*_*k*_. It then compares the value of *Q*_*k*_ with minimum TXOP limit, *TXOP*_*min*_ and maximum TXOP limit *TXOP*_*max*_. The range of the TXOP limit is calculated using the IEEE802.11e standard and are in between 32 *μ*sec and 8160 *μ*sec. The minimum threshold in bits, Γ_*min*_, and the maximum threshold in bits, Γ_*max*_, are then computed according to the Eqs (10) and (11), respectively.

$$\begin{array}{c} \Gamma_{min}=(TXOP_{min}*R_{k}). \end{array}$$ (10)

$$\begin{array}{c} \Gamma_{max}=(TXOP_{max}*R_{k}). \end{array}$$ (11)

**Fig 3:**
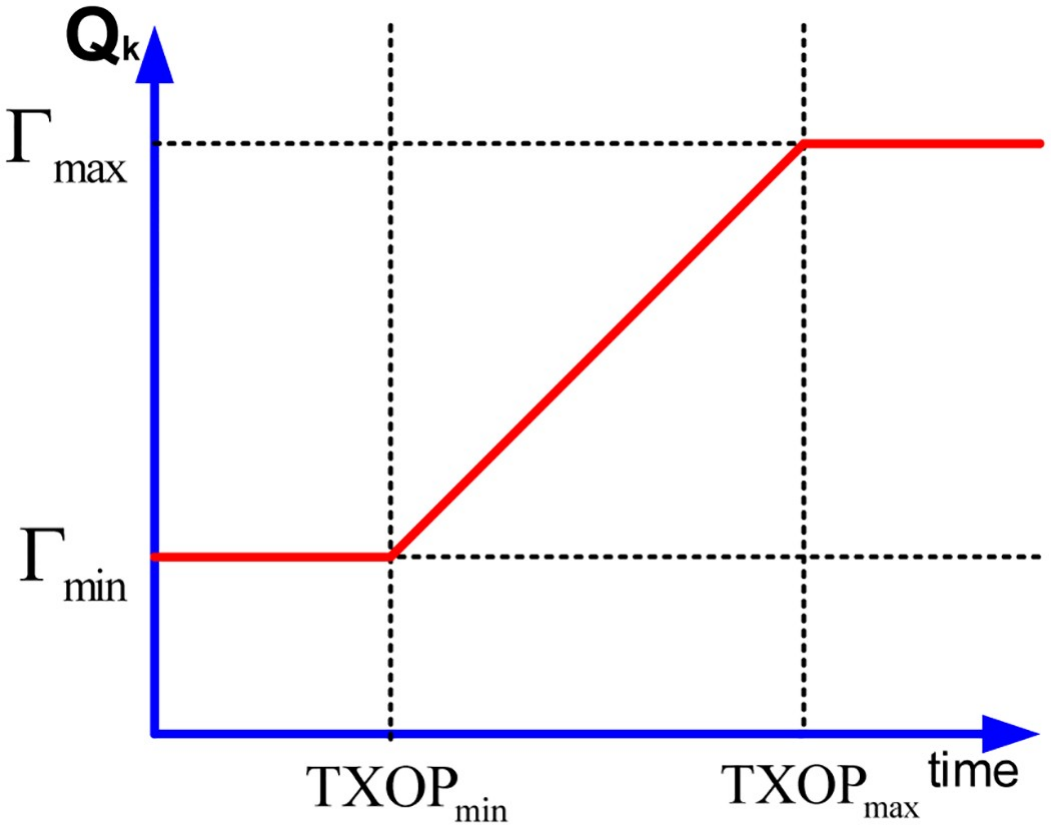
The EDTXOP limit allocation scheme.

The intention behind computing Γ_*min*_ and Γ_*max*_ is to enable their comparison with the current aggregated queue size *Q*_*k*_. Algorithm 1 shows the steps of the EDTXOP mechanism, which allocates the TXOP limit dynamically by the QAP for each traffic queue of the QSTA. Here, in case when *Q*_*k*_ is greater than or equal to Γ_*max*_, then the current assigned TXOP limit, *TXOP*_*k*_, is set to the maximum TXOP limit. In contrast, if *Q*_*k*_ is lower than Γ_*min*_, then *TXOP*_*k*_ is set to the minimum TXOP limit. Otherwise, if *Q*_*k*_ value is in between Γ_*max*_ and Γ_*min*_ values, then the *TXOP*_*k*_ is computed based on the *Q*_*k*_ at that time instant as shown in the algorithm. The dynamic TXOP limit allocation scheme is shown in Fig 3.

**Algorithm 1** EDTXOP limit allocation

1: **function** initiatlization

2:  *TXOP*_*max*_ = *min* $\left(\frac{\sum_{t=1}^{n}F_{k}}{R_{k}}\right)$

3:  *TXOP*_*min*_ = *max* $\left(\frac{F_{k}}{R_{k}}\right)$

4:  Γ_*min*_ = (*TXOP*_*min*_ * *R*_*k*_)

5:  Γ_*max*_ = (*TXOP*_*max*_ * *R*_*k*_)

6: **end function**

7: **function** TXOP_limit_allocation

8:  **if** (*Q*_*k*_ ≥ Γ_*max*_) **then**

9:   *TXOP*_*k*_ = *TXOP*_*max*_

10:  **else if** (*Q*_*k*_ ≤ Γ_*min*_) **then**

11:   *TXOP*_*k*_ = *TXOP*_*min*_

12:  **else**

13:   $TXOP_{k}=(\frac{Q_{k}*TXOP_{max}}{\Gamma_{max}})or(\frac{Q_{k}*TXOP_{min}}{\Gamma_{min}})$

14:  **end if**

15: **end function**

### Simulation setup

In this section, the simulation parameters utilized in the proposed work are described. For the EDTXOP scheme, the EDCA service differentiation protocol is employed for IMM traffics enabling access to the channel based on assigned priorities. The simulation area considered here is 1000 m x 1000 m. The number of mobile nodes representing clients is set to 30. Furthermore, a single static access point (AP) is considered as the receiver. The beacon interval has been fixed to 100 msec based on the IEEE standard [10]. AODV routing protocol is utilized to select the best path and the random waypoint mobility model is used for the node’s movement in order to reflect the real-world scenarios. The system parameters used in EDTXOP mechanism follows the IEEE 802.11e standard [11] and are summarized in Table 2.

**Table 2:** System parameters [11].

Parameter	Value
Short Intra Frame Space (SIFS)	16 *μ*sec
Acknowledge Frame Size ACK	14 Bytes
Frame Header Size *H*_*k*_	34 Bytes
Maximum MSDU Size	2304 Bytes
Maximum TXOP limit	8160 *μ*sec
Minimum TXOP limit	32 *μ*sec

For the performance evaluation of the EDTXOP mechanism over the network, the traffic loads are varied into two cases. Firstly, keeping a constant value of the packet arrival rate (50 packets per second) and varying the traffic load by considering different packet sizes: 256, 512, 1024, 1536, 2048, 2560 and 3072 Bytes. For the second case, the packet size is fixed to 1600 Bytes and the traffic load is varied by considering different packets arrival rates: 8, 16, 32, 48, 64, 80 and 96 packets per second. The EDTXOP mechanism is simulated by Qualnet 5.1 network simulator [36]. Table 3 shows the simulation parameters set for the EDTXOP scheme.

**Table 3:** Simulation parameters of the EDTXOP scheme.

Parameter	Value
Simulation Area	1000 m x 1000 m
Simulation Time	100 sec
Propagation model	Two-ray ground [33]
Shadowing model	Constant
MAC protocol	IEEE802.11e
Radio Type	IEEE802.11b
PHY data rate	11 Mbps
Routing protocol	AODV [34]
Antenna Type	Omnidirectional
Mobility model	Random Waypoint [35]
Data Traffic	CBR

### Performance metrics

The performance of the EDTXOP scheme is evaluated in terms of throughput, PDR, average ETE delay, and average jitter. These performance metrics are described next.

- **Throughput (bits/sec)**: It is defined as the ratio of the total amount of successfully delivered packets in bits to the total time taken between the first packet sent and the last packet received.
  $$\begin{array}{c} Throughput=\frac{N_{r}*S_{bits}}{t_{tot}}, \end{array}$$ (12)
  where *N*_*r*_ is the total number of packets received at the receiver side. *S*_*bits*_ is the size of each packet in bits and *t*_*tot*_ is the total time elapsed between the first packet sent and the last packet received.
- **PDR (%)**: PDR is defined as the ratio of total number of received packets at the receiver side to the total number of packets sent from the sender. PDR is computed as follows:
  $$\begin{array}{c} PDR=\frac{N_{r}}{N_{s}}*100, \end{array}$$ (13)
  where *N*_*s*_ is the total number of packet sent from the sender side.
- **Average ETE Delay (sec)**: The total time taken by the packet, that is generated at the sender, to reach the receiver is referred to as ETE delay. For instance, if *t*_*s*_ is the timestamp when the packet is generated at the sender and *t*_*d*_ is the timestamp when the packet reaches the receiver. The ETE delay is then computed as follows:
  $$\begin{array}{c} \text{ETE}\;\text{Delay}=t_{d}-t_{s} \end{array}$$ (14)
  Average ETE delay can then be computed as the ratio between the sum of ETE delays of all the packets that are received by the receiver and number of packets received at the receiver and is as follows:
  $$\begin{array}{c} \text{Average}\;\text{ETE}\;\text{delay}=\frac{\sum_{n=1}^{N_{r}}\text{ETE}\;{\text{delay}}_{n}}{N_{r}}, \end{array}$$ (15)
- **Average Jitter (sec)**: Jitter represents the variation of time between current packet reception and previous packet reception at the receiver. For instance, if the the time taken by the current packet to reach the receiver is *t*_*c*_ and the time taken for the previous packet to reach receiver is *t*_*p*_ then,
  $$\begin{array}{c} Jitter=t_{c}-t_{p} \end{array}$$ (16)
  Average Jitter can then be calculated as follows:
  $$\begin{array}{c} \text{Average}\;\text{Jitter}=\frac{\sum_{n=1}^{N_{r}-1}{\text{Jitter}}_{n}}{N_{r}-1}, \end{array}$$ (17)

## Results and discussion

The performance of EDTXOP scheme is compared with the performance of the static TXOP (STXOP) [11], TBD-TXOP [13, 16]. The simulation results for the two cases are explained next.

### Impact of the increasing traffic load based on the variation of packet sizes

In this section, the traffics load is increased from 100 to 1200 kbps with respect to the variation of different packet sizes. The performance is evaluated in terms of throughput, PDR, average ETE delay, and average jitter.

#### Throughput analysis

**Fig 4:**
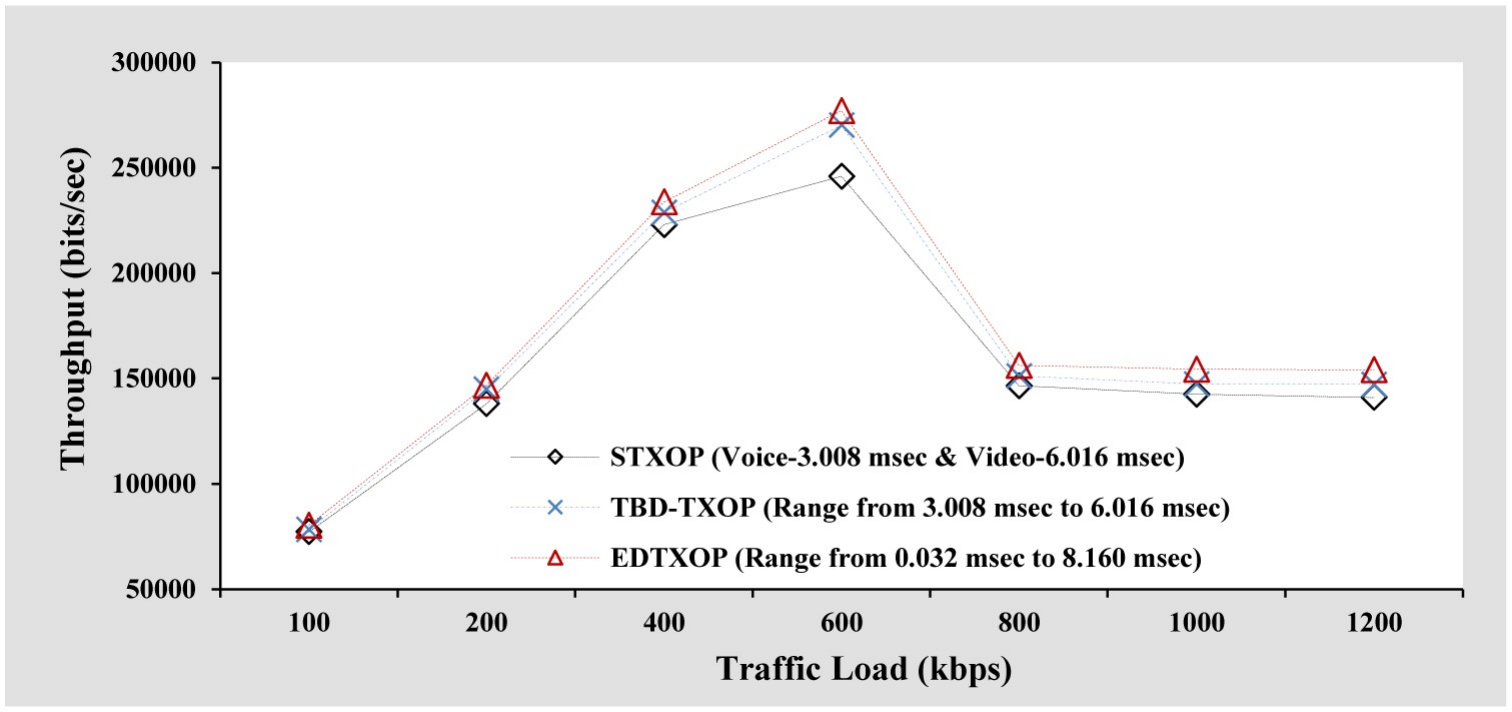
Throughput analysis based on increasing packet size.

Fig 4 represents the performance comparison of throughput for high priority traffic among STXOP, TBD-TXOP and EDTXOP schemes. A sharp increase is observed in the throughput when the traffic load increases from 100 kbps to 600 kbps for all mechanisms. This is due to the successful reception of a large number of bits by the receiver. Further increase in the traffic load results in a decrease in throughput and the throughput remains consistent from 800 kbps to 1200 kbps. The reason being heavy traffic load, causing a small number of bits reception at the receiver. Although, TBD-TXOP assigns the TXOP limit dynamically, EDTXOP scheme achieves better throughput. The improved throughput in the case of the EDTXOP scheme is due to the regulation of the dynamic TXOP limit within the range of threshold limit for high priority traffic according to the demand of the current traffic load.

#### PDR analysis

**Fig 5:**
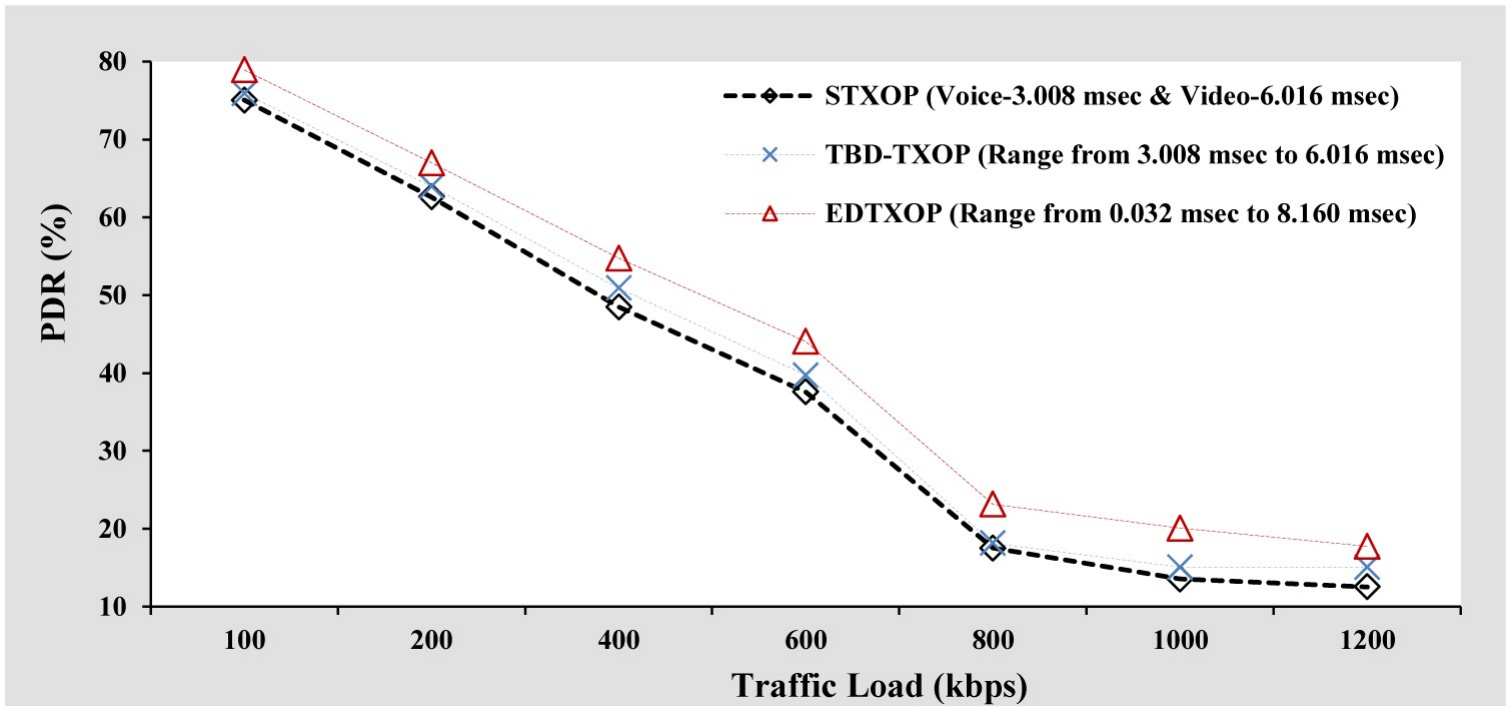
PDR analysis based on increasing packet size.

From Fig 5, it can be observed that PDR decreases with increasing traffic load implying high packet loss. Although PDR decreases gradually when the traffic load is varied from 100 kbps to 600 kbps, a point to notice is that with increasing traffic load, the number of bits received at the receiver increases. This is because the packet arrival rate for this case is fixed and the only way to increase the traffic load is to increase the packet size. Therefore, at high traffic load, even though the number of packets received at the receiver is less, the increase in the packet size results in the higher number of bits received at the receiver. Another point to notice is that the EDTXOP mechanism offers superior PDR performance when compared to STXOP and TBD-TXOP at all traffic load conditions.

#### Average ETE delay analysis

**Fig 6:**
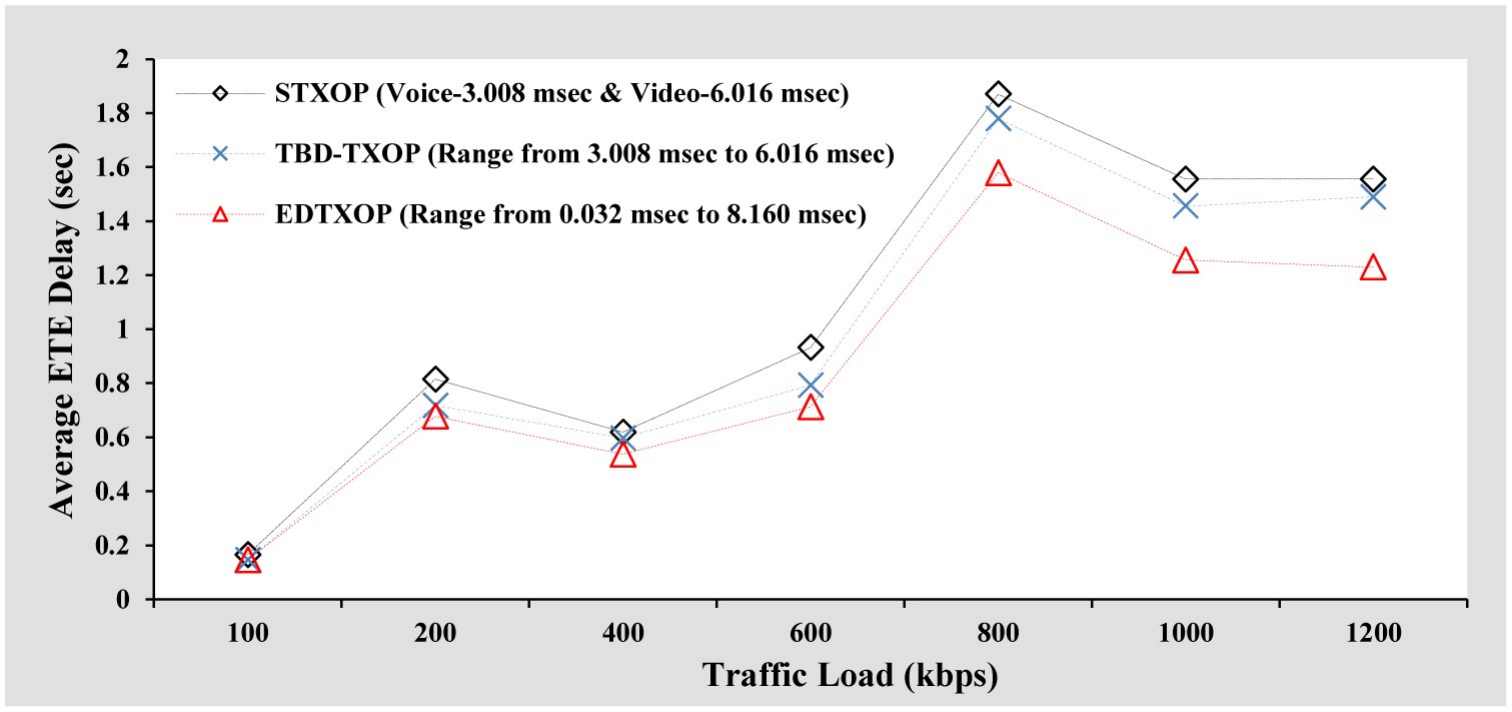
Average ETE delay analysis based on increasing packet size.

As can be seen in Fig 6, the average ETE delay increases with rise in the traffic load. This is because, with increasing packet size, the time taken to transmit the packets from the sender increases, thereby contributing to increased average ETE delay. Another point to notice is that when the traffic load is varied from 800 kbps to 1200 kbps, the average ETE delay decreases slightly. The reason being a large number of packets dropped due to heavy traffic load. The EDTXOP mechanism results in the lowest average ETE delay for high priority traffic when compared to STXOP and TBD-TXOP. In addition, as the traffic load increases the average ETE delay differences of EDTXOP with STXOP and TBD-TXOP increase. This is due to the EDTXOP scheme’s optimal utilization of channel bandwidth by assigning an appropriate variable TXOP limit.

#### Average jitter analysis

**Fig 7:**
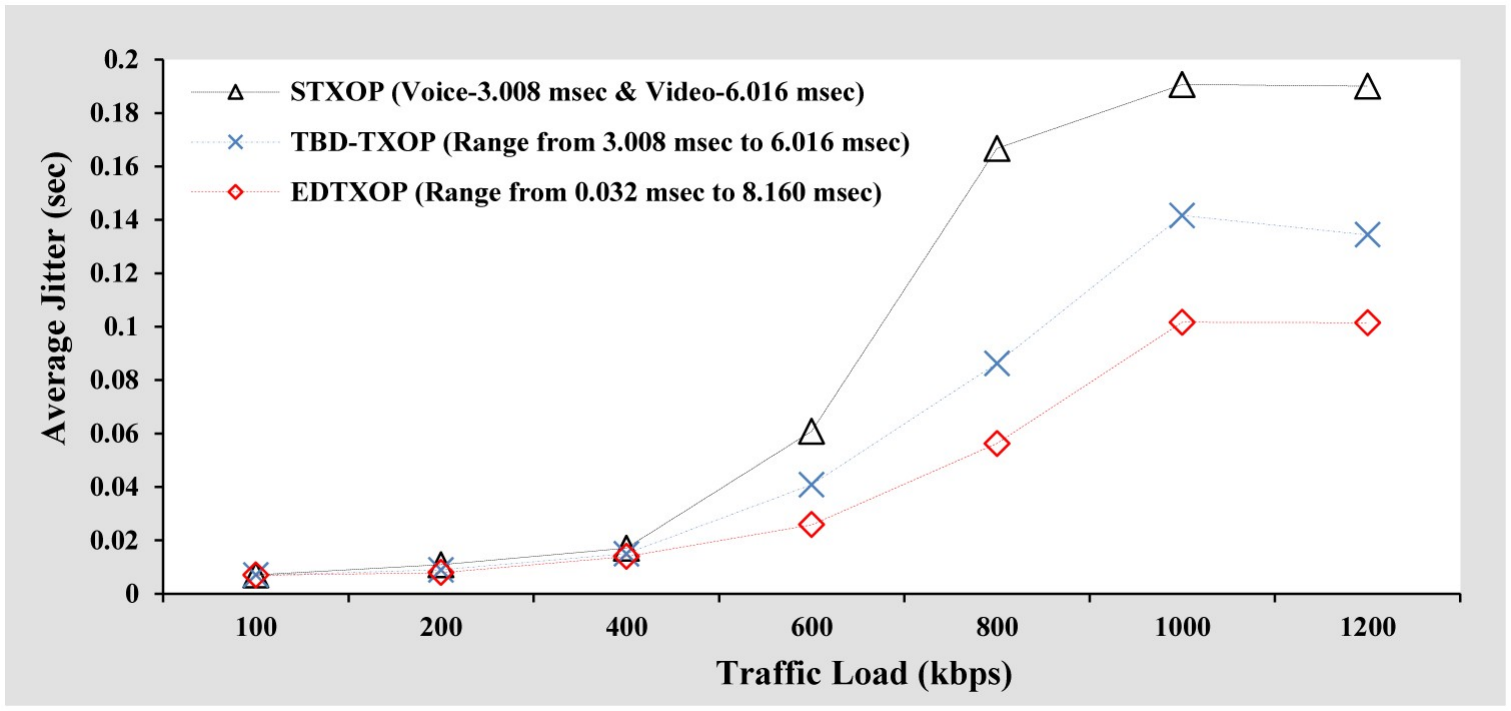
Average jitter analysis based on increasing packet size.

Fig 7 compares the average jitter performance of EDTXOP scheme with the performance of STXOP and TDB-TXOP. It can be observed that until the traffic load reaches 400 kbps, the performance difference among the schemes is minimum and is due to the low traffic load and collision probability. However, with further increase in traffic load, the average jitter increases and is due to increased packet collisions as well as the high number of retransmissions required to transfer the traffic through the channel. STXOP scheme results in high average jitter due to the fixed TXOP limit and unnecessarily occupies the channel for a constant duration in case of high priority IMM traffics even when the low priority traffics are waiting to access the channel. On the other hand, the EDTXOP scheme shows the lowest average jitter than the rest because it assigns the variable TXOP limit seamlessly and reduces the retransmission attempts. In the case of low priority traffic, the EDTXOP scheme also attains the lowest average jitter because it occupies the channel with a variable duration of time.

### Impact of the increasing traffic load based on the variation of the packets arrival rate

In this section, different packet arrival rates (8, 16, 32, 48, 64, 80, and 96 packets per second) are utilized to vary the traffic load while keeping the packet size constant. As observed in the throughput analysis in Fig 4 of the previous section, a packet size of 1536 Bytes (at 600 kbps) offers the best throughput. Therefore, in order to fix the packet size for this case, the aim was to select a packet size that is very close to the optimal packet size (1536 Bytes). For this reason, a packet size of 1600 Bytes was selected so that the packet arrival rates of 8, 16, 32, 48, 64, 80 and 96 packets per second results in the traffic loads of 100 kbps, 200 kbps, 400 kbps, 600 kbps, 800 kbps, 1000 kbps, and 1200 kbps, respectively.

#### Throughput analysis

**Fig 8:**
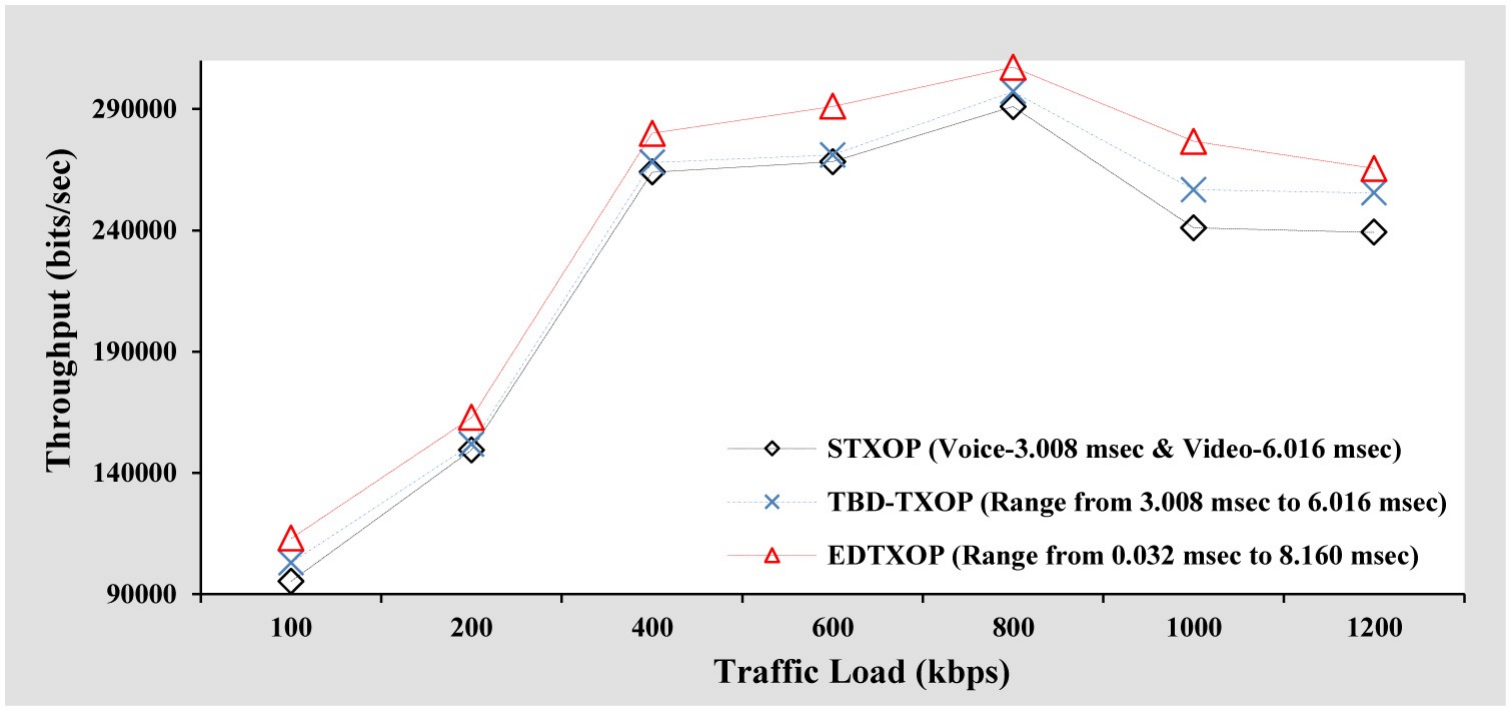
Throughput analysis based on increasing packets arrival rate.

Fig 8 illustrates the throughput comparison of EDTXOP scheme with STXOP and TBD-TXOP by varying the traffic load in terms of changes in the packet generation rate from clients. It can be observed that throughput increase gradually with increasing traffic load and is due to the successful reception of a large number of bits by the receiver. In addition, the EDTXOP scheme achieves better overall throughput when compared to STXOP and TBD-TXOP which is due to the EDTXOP scheme’s enhanced regulation of dynamic TXOP limit within the range of threshold limit and is according to the demand of current traffic load. Another point to notice is that the peak of throughput in this analysis is at 800 kbps when compared to 600 kbps peak in the throughput analysis in Fig 4 of the previous section. In addition, the overall throughput performance of this scenario is better than the throughput performance in Fig 4 of the previous section. The main reason for the throughput performance improvement is the selection of appropriate packet size (1600 Bytes) which is close to optimal packet size (1536 Bytes). A small packet size has a large share of overhead which implies even after utilization of channel, fewer bits are received by the receiver due to unnecessary overhead. On the other hand, although a large packet size has a small share of overhead, packet drops results in loss of a large chunk of bits, thereby resulting in less number of bits received at the receiver. Therefore, a packet size of 1600 Bytes provides an optimum trade-off between unnecessary overhead and loss of a large number of bits, thereby offering the best throughput performance.

#### PDR analysis

**Fig 9:**
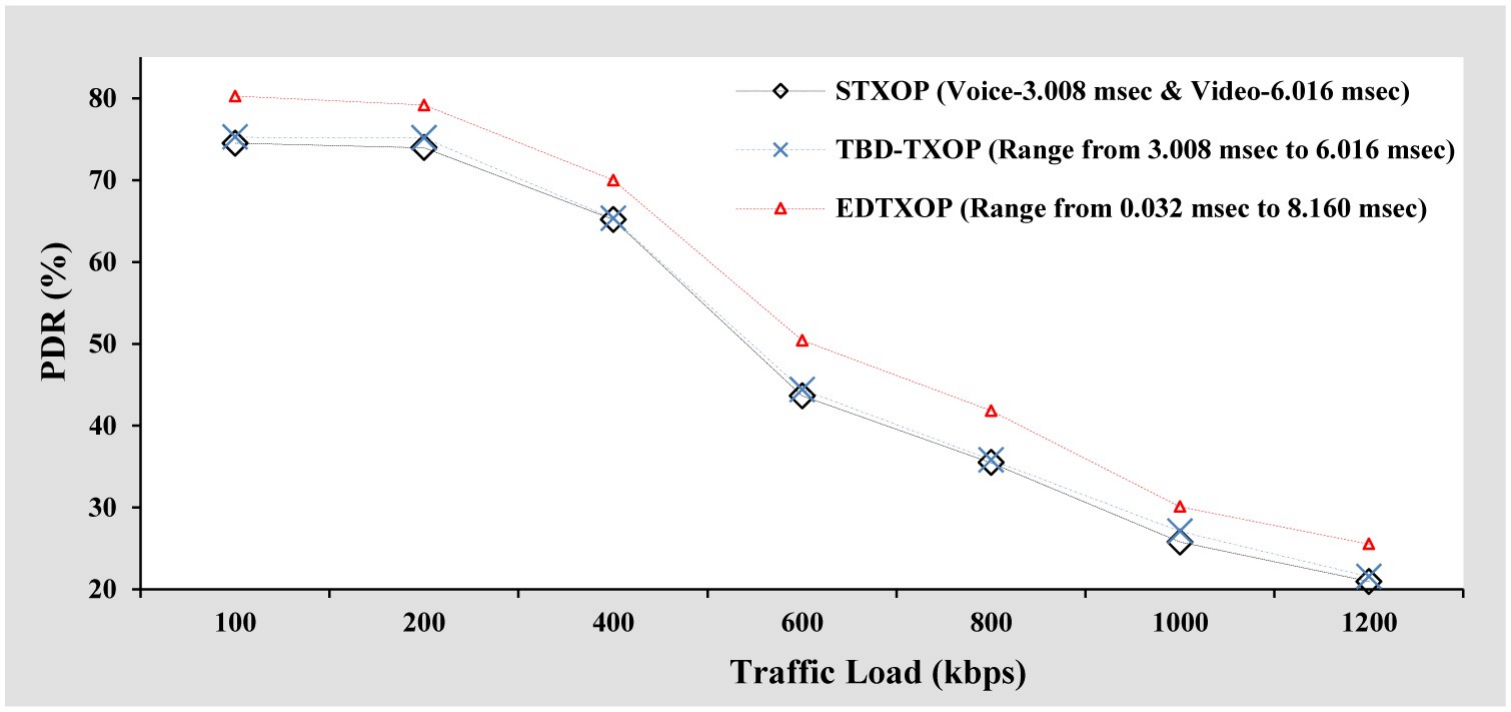
PDR analysis based on increasing packets arrival rate.

Fig 9 shows the PDR performance comparison of EDTXOP scheme with STXOP and TBD-TXOP by increasing the traffic load in terms of variation in the packet generation rate from clients. It can be observed that with rising traffic load, the PDR performance for all the schemes degrades, referring to high packet loss. Apart from this, the EDTXOP scheme outperforms STXOP and TBD-TXOP by offering high PDR at all traffic load conditions. The reason being the optimal utilization of channel bandwidth by assigning appropriate variable TXOP limit while also reducing the packet drops compared to the other two schemes.

#### Average ETE delay analysis

**Fig 10:**
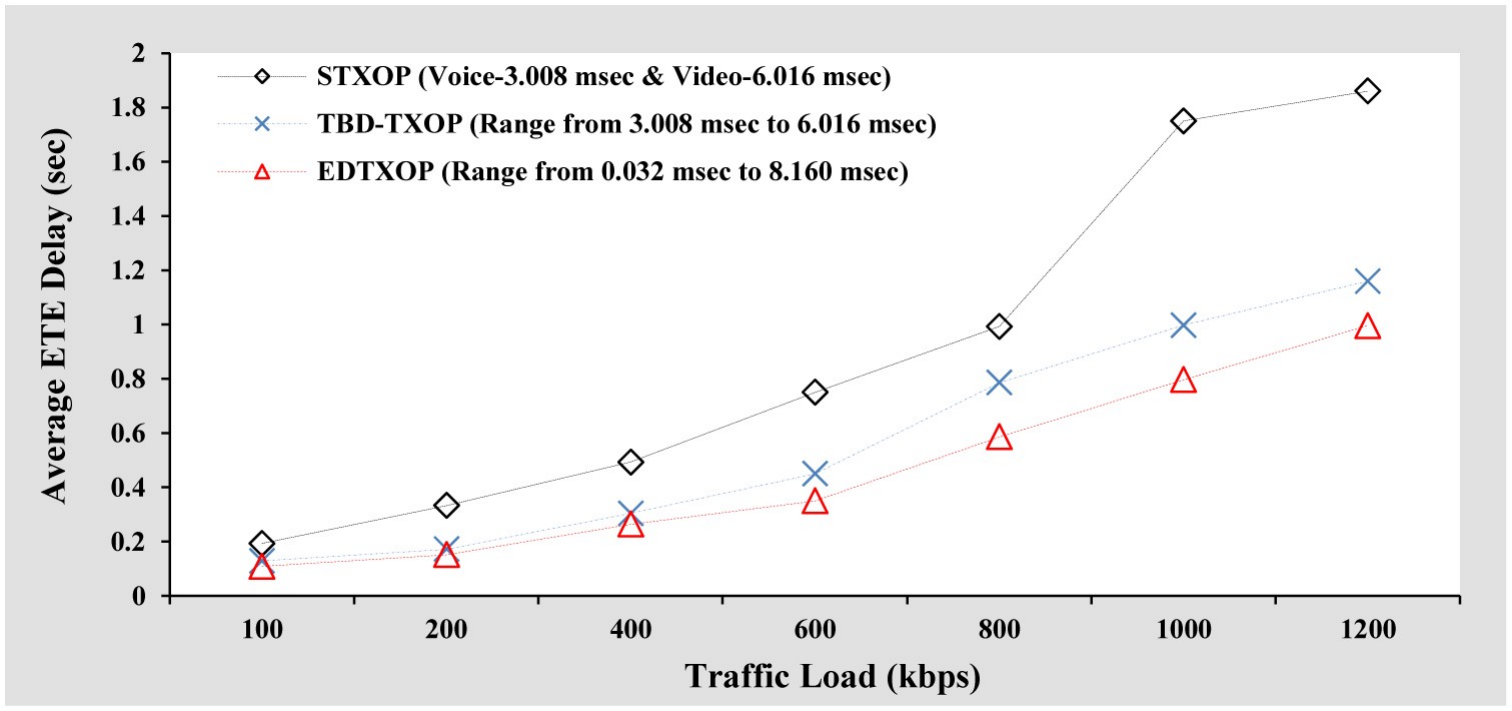
Average ETE delay analysis based on increasing packets arrival rate.

Fig 10 represents the average ETE delay performance comparison of the EDTXOP scheme with the performance of STXOP and TBD-TXOP by varying the traffic load in terms of changes in the packet generation rate from clients. It can be observed that with rising traffic load, the average ETE delay of all the schemes increases. This is because, with an increasing number of packets, the total transmission time and queuing delay increase, thereby contributing to a high average ETE delay. Another observation is that the EDTXOP scheme offers the lowest average ETE delay when compared to the rest. In addition, as the traffic load increases, the average ETE delay performance differences of the EDTXOP scheme with STXOP and TBD-TXOP increase. This is because of the EDTXOP scheme’s appropriate assignment of variable TXOP limit to utilize the channel bandwidth optimally.

#### Average jitter analysis

**Fig 11:**
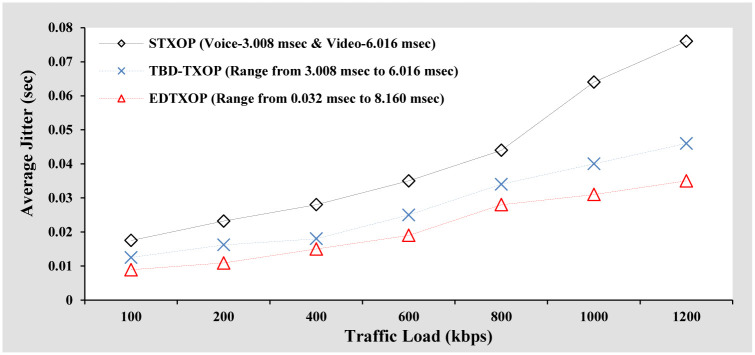
Average jitter analysis based on increasing packets arrival rate.

Fig 11 shows the average jitter performance comparison of the EDTXOP scheme with STXOP and TDB-TXOP by increasing the traffic load in terms of variation in the packet generation rate from clients. As can be seen in the figure, with a rise in the traffic load, the average jitter increases for all the schemes and is mainly due to two reasons. The first reason is a large number of retransmissions necessary to allow packet exchange through the channel while the second reason being high packet collisions. The EDTXOP scheme attains the lowest average jitter for high priority traffic as it assigns the TXOP limit dynamically based on the requirements of the current traffic load while also utilizing the channel bandwidth appropriately. Furthermore, it reduces the re-transmission attempt and achieves the lowest variation of time compared to other schemes.

### Key observations

From the above-discussed simulation results, the key observations can be summarized as follows:

- The traffic load was varied in terms of two cases. Firstly, the packet size was varied while keeping the packet arrival rate constant. Secondly, the packet arrival rate was varied while keeping the packet size constant. For both cases, it was observed that an increase in traffic load results in decreased PDR, increased average ETE delay, and average jitter. Apart from this, the key finding from the throughput analysis of the first case is the optimal packet size selection for the second case. The impact of the optimal packet size selection can be observed in the throughput analysis of the second case, where the throughput peak remains for a higher traffic load when compared to the throughput peak of the first case.
- STXOP, after reaching a specific traffic load (600 kbps for the first case and 800 kbps for the second case), results in a sharp increase in average ETE delay and average jitter. The reason being fixed TXOP limit, where irrespective of traffic load of the high priority traffic a constant duration is assigned. This further results in unnecessary waiting of low priority traffic even though there is no high priority traffic available to transfer, thereby leading to the wastage of channel bandwidth. Therefore, contributing to increased average ETE delay and average jitter.
- Finally, the proposed EDTXOP scheme outperforms STXOP and TBD-TXOP for all the evaluated performance metrics and both cases. This is due to the EDTXOP scheme’s enhanced regulation of dynamic TXOP limit based on the requirement of the current traffic load and accurate allocation of dynamic TXOP limit by effective computation of the current queue size.

## Conclusion

This article proposes the EDTXOP scheme which offers a dynamic resource allocation solution to manage frequently varying traffic load over WCN while also meeting the QoS requirements for IMM traffic flows. In this work, two simulation scenarios have been carried out by varying packet size and packet arrival rate while keeping one of these parameters fixed for each scenario. From the first scenario, an optimal packet size has been found and used as the basis for the packet size selection in the second scenario. The simulation results evaluation shows that the proposed EDTXOP scheme achieves the overall performance gains in the range of 4.41%—8.16%, 8.72%—11.15%, 14.43%—32% and 26.21%—50.85% for throughput, PDR, average ETE delay and average jitter, respectively when compared to STXOP and TBD-TXOP. Thus, highlighting the performance superiority of the EDTXOP scheme over the rest. Analyzing the compatibility of the EDTXOP scheme in networks other than WCN is considered as part of the future work.
